# Advanced Freeze-Drying Modeling: Validation of a Sorption-Sublimation Model

**DOI:** 10.1021/acsomega.5c01665

**Published:** 2025-04-18

**Authors:** Alex Juckers, Andreas Potschka, Jochen Strube

**Affiliations:** Institute for Separation and Process Technology, Clausthal University of Technology, Clausthal-Zellerfeld 38678, Germany

## Abstract

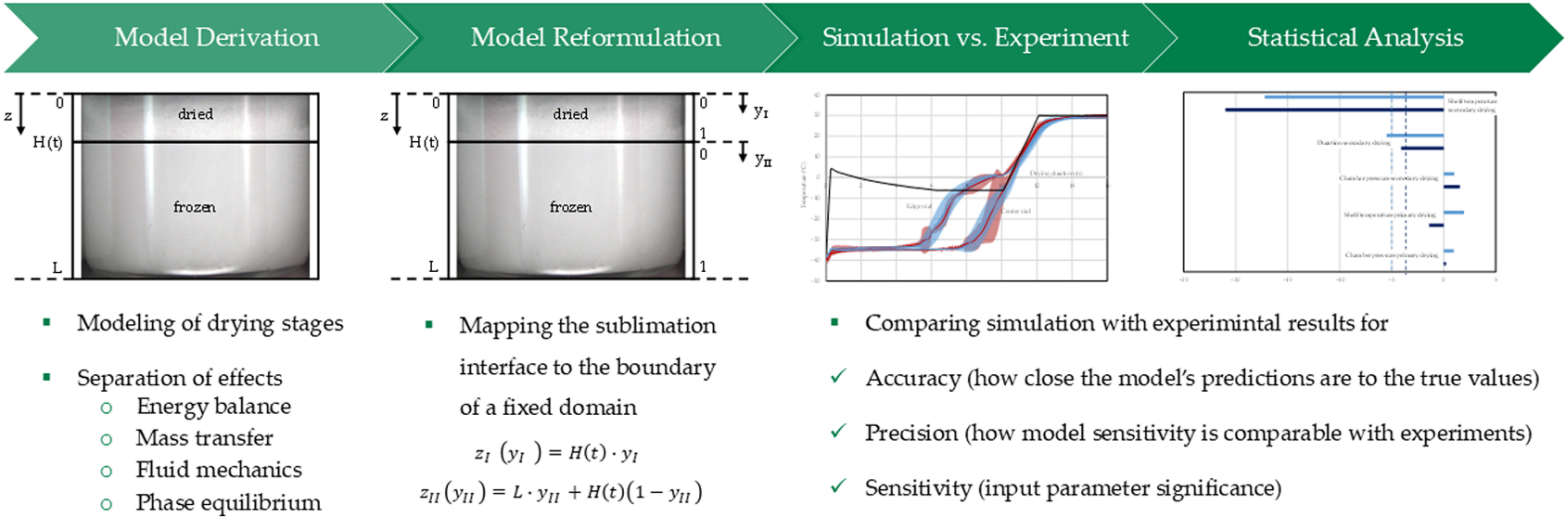

Modeling freeze-drying is crucial due to the complex interplay of heat and mass transfer, which significantly impacts product quality and process efficiency. Traditional experimental approaches can be time-consuming and resource-intensive, making rigorous modeling an essential tool for optimization. Sorption-sublimation models incorporate the dynamic nature of the drying steps by accurately describing heat and mass transfer, enabling precise calculation of product temperature and residual moisture. Here, the moving boundary is mapped to the boundary of a fixed domain by the introduction of two new coordinates. Simulation results are validated by the approach from Sixt et al. The model shows good agreement with experimental data, with deviations reduced to as low as 3.9%. This represents a significant improvement over previous models, such as the pseudostationary approach, which exhibits deviations up to 42.2% for edge vials at the end of primary drying. For residual moisture, the experiment and simulation show similar error margins of 7% and the simulation deviates by only 8% from the experimental value. The model’s accuracy and precision offer valuable insights for optimizing process parameters, ultimately enhancing product quality and reducing development costs. The successful validation against experimental data shows the model’s potential as a robust tool for predicting process behavior in vial lyophilization, paving the way for its application in both research and industrial settings.

## 1 Introduction

Lyophilization, also known as freeze-drying, is a key technology in the pharmaceutical industry to stabilize sensitive biological products such as vaccines, proteins, and other biopharmaceutical preparations.^1−3^ The process involves three steps:

- **Freezing**: Transformation of liquid water into solid ice, vitrification, or crystallization of solutes.
- **Primary drying**: Removal of solid ice through sublimation under vacuum, creating a porous cake consisting of solutes and bound water.
- **Secondary drying**: Removal of bound water through desorption, achieving low residual moistures.

This multistep process requires precise control and optimization to ensure product quality while maximizing efficiency and cost-effectiveness. During the drying steps, the product temperature must be kept under a formulation-dependent critical temperature in order to maintain the physical form of the lyophilized product.^4^ However, the product temperature is not directly controlled but rather established through shelf temperature and chamber pressure,^5,6^ making purely experimental selection of process parameters challenging, time-consuming, and expensive. As part of the regulatory required Quality-by-Design (QbD) approach, physicochemical process modeling can be leveraged to deepen process knowledge, explore the design space, and perform process optimization, thereby reducing experimental effort.^7^

The main process steps freezing and primary and secondary drying can be modeled to predict operation ranges and performance for process design and optimization as well as scale-up and manufacturing scale process control. However, there is a trade-off between accuracy and complexity. The more complex the mathematical description, the more computationally intensive the simulation and the more time it takes to determine the model parameters. Physicochemical based mechanistic modeling has proven to be the most efficient in terms of validated accuracy (how close the model’s predictions are to the true values) and precision (how sensitivity of the model is comparable within the sensitivity of experiments) for quantitative predictive power with an efficient approach to model parameter determination based on a few clearly defined laboratory scale experiments.^8−10^ According to this approach, the lyophilization process must be described mechanistically, i.e., based on physicochemical fundamentals, while separating the different fundamental phenomena, such as fluid dynamics, mass transfer, phase equilibrium, and energy balance. This allows scale-up, as only fluid dynamics is scale dependent. Since the fluid dynamics of equipment at different scales from laboratory to production are known, the other model parameters can be determined a priori at the small laboratory scale. In general, physical properties have to be determined experimentally for each new chemical component system.^11−14^ Nevertheless, physical property databases are available, e.g., DETHERM,^15^ Dortmund Data Bank,^16^ and NIST,^17^ as well as many in-house company documentations. Only mechanistic, physicochemical models allow comparison and evaluation with different model parameters across different component systems and groups to learn from existing data and predict new data. Machine learning, especially artificial neural networks, are a great help with increasing potential to reduce the number of experiments.^18^

The application of detailed physicochemical modeling has been discussed^19^ in comparison to finite element approaches^20^ and short-cut macroscopic models.^21^ Consequently, an experimentally validated concept for fast process design and scale-up from laboratory to pilot and manufacturing scale has been proposed in combination with the evaluation of various available process analytical technology (PAT) sensors.^22−26^ Most of the effort has been directed at modeling the drying steps, as primary drying is by far the longest step in most processes and offers significant potential for optimization. However, modeling of the freezing step has been increasingly reported.^27−31^

For the drying phases, two models have been mainly utilized for the process description: pseudostationary and sorption-sublimation model. The first one is a simplification of the latter one. We begin with the pseudostationary model. In this model, pseudostationary conditions are assumed for the heat and mass transfer of the primary drying phase. This is reasonable due to the slow kinetics of the sublimation process. All of the heat supplied by the shelf is used for sublimation. Heat accumulation in both phases is neglected.^32^

The coupled heat and mass transfer is described by
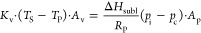
1where *K*_v_ is the vial heat transfer coefficient, *T*_s_ is the shelf temperature, *T*_p_ is the product temperature, *A*_v_ is the outer cross-sectional area of the vial, Δ*H*_subl_ is the sublimation enthalpy, *R*_P_ is the dry layer resistance, *p*_i_ is the partial vapor pressure on the sublimation interface, *p*_c_ is the chamber pressure, and *A*_p_ is the inner cross-sectional area of the vial. The left-hand side of the equation describes the effective heat input from the shelf into the product, while the right-hand side describes the amount of heat used to sublimate the ice. Since heat accumulation is neglected, the heat conduction within the frozen layer can be described as
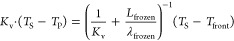
2where *L*_frozen_ is the length of the frozen area, λ_frozen_ is the heat conductivity of ice, and *T*_front_ is the sublimation interface temperature.

The sorption-sublimation model is capable of calculating the transient behavior during both drying phases at discrete points in the product.^5,32,33^ For the calculation, heat and mass balances are formulated for the dried and frozen zones as well as for the sublimation interface. The heat balance of the dried layer (layer I) can be described as
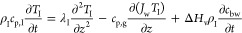
3where *T*_I_ (*t, z*) is the dried layer temperature at time *t* and axial coordinate *z*; ρ_I_, *c*_p,I_, and λ_I_ are the density, heat capacity, and thermal conductivity of the dried layer, respectively; *c*_p,g_ is the gas heat capacity; *J*_w_ (*t*, *z*) is the mass flux of water at time *t* and axial coordinate *z*; Δ*H*_v_ is the vaporization enthalpy; and *c*_bw_ is the concentration of sorbed water. Radial temperature variations are neglected. The left term describes the heat accumulation of the dried layer, the first term on the right describes the heat flow into the dried region, the second describes the heat transport by convection of the gas, and the last describes the heat required to desorb bound water. Time-scale analysis of these heat transfer effects revealed that the gas convection contribution can be neglected since it is much faster than heat conduction of the porous matrix.^34^

The frozen layer (layer II) can be described analogously as
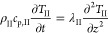
4where *T*_II_ (*t*, *z*) is the frozen layer temperature at time t and axial coordinate *z*; and ρ_II_, *c*_p,II_, and λi are the density, heat capacity, and thermal conductivity of the frozen layer, respectively. The left term describes the heat accumulation in the frozen phase, and the right term describes the heat transport by conduction.

The sublimation boundary layer is located at the contact point between the frozen and dried regions and moves downward toward the bottom with ongoing primary drying duration. Here, the heat balance can be calculated as
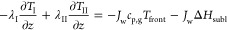
5where *T*_front_ (*t*) is the sublimation interface temperature at time *t*. The two terms on the left-hand side model the heat flows from the two regions to the sublimation interface. The first term on the right describes the heat transport by convection, and the last term describes the heat removed by the sublimation of the ice.

The heat transfer equations are completed with two Neumann-type and one Dirichlet boundary conditions. At the bottom (*z* = *L*), conduction from the shelf must be considered while radiation is the main heat transfer mechanism on the top of the dried layer (*z* = 0). On the sublimation front, the temperatures of both phases are equal
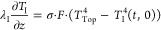
6
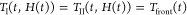
7
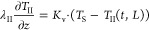
8where σ is the Stefan–Boltzmann constant, *F* is the view factor for radiative heat transfer, and *T*_Top_ is the temperature of the radiating body.

During freeze-drying, ice is sublimated and bound water is desorbed. The mass balance of the dried layer can thus be represented as
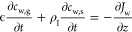
9where ε is the porosity, *c*_w,g_ (*t*, *z*) is the concentration of water vapor at time *t* and axial coordinate *z*, and *c*_w,s_ (*t*, *z*) is the mass share of sorbed water at time *t* and axial coordinate *z*. The first left-hand term describes the change in the water content in the gas phase, and the second term describes the change in the water content in the solid matrix. The right-hand term models the water mass flow out of the system, which can be described by
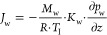
10where *M*_w_ is the molecular weight of water, *R* is the gas law constant, *K*_w_ is the Knudsen flow mass transfer coefficient, and *p*_w_ (*t*, *z*) is the partial pressure of water at time *t* and axial coordinate *z*. *K*_w_ is calculated as the inverse of the derivative of *R*_p_ relative to the dry layer height.^33^

The axial position of the sublimation interface, *H*(*t*), can be tracked by its velocity^35^
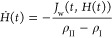
11

Secondary drying begins as soon as the sublimation front passes. Bound water is removed from the product by desorption. Desorption consists of different steps:^36^ molecular diffusion of water to pore surface, phase transition at solid–vapor interface, and convection through the dried layer and subsequently through the vial headspace to the ice condenser. Extensive research by Pikal et al. showed that phase transition on the solid–vapor interface is the rate determining factor during secondary drying.^37^

The temperature-dependent desorption kinetic of the bound water is calculated with the following equations, where the desorption time follows an Arrhenius relationship^38^
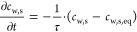
12
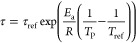
13where τ is the desorption time, *c*_w,s,eq_ is the equilibrium water content, τ_ref_ is the value of time constant at reference temperature, *E*_a_ is the activation energy, and *T*_ref_ is the reference temperature. With increasing product temperature, the desorption time is shortened, facilitating the desorption process.

The water content *c*_w,s,eq_ is calculated by the Guggenheim–Anderson–Boer (GAB) equation for the sorption isotherm, which reads as
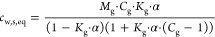
14with *M*_g_, *K*_g_, and *C*_g_ as product-dependent constants and α as the water activity. During secondary drying, the vapor composition changes from mainly water to inert gas. Water activity is calculated as a quotient between the partial pressure of water and equilibrium vapor pressure. The water vapor pressure can be calculated with the binary gas transport model.^39^

To solve the mass balances, the partial pressure of water is set equal to the chamber pressure at *z* = 0 and at the sublimation interface *H*(*t*) during primary drying ice and water vapor are in thermal equilibrium, allowing the calculation of the temperature-dependent vapor pressure with the new sublimation pressure equation.^40^

The equations of the sorption-sublimation model can be extended from mono- to multidimensional description of the process. However, due to the increase in complexity, they are not suitable for real-time implementation.^33,41−43^Table 1 lists several modeling approaches with the used product solution, heat transfer mechanism, and area of application taken from literature.

**Table 1:** Various Modeling Approaches and Their Areas of Application^a^

	type	product	heat transfer	application
(44)	Dyn	skim milk	vial bottom: radiation and gas conduction	calculation of the dynamic behavior of primary and secondary drying (*T*_P_, *w*_H2O_, *t*_Drying_)
			product surface: radiation	
(21)	Pse	PVP	vial bottom: contact, gas conduction, and radiation	process control and optimization
		mannitol		
(45)	Dyn	0.2 kg/L cloxacillin	vial bottom: gas conduction and radiation	calculation of the dynamic behavior of primary and secondary drying (*T*_P_, *w*_H2O_, *t*_Drying_)
		sodium salt	product surface: radiation, gas conduction and convection	
		skim milk		
(46)	Pse	water	vial bottom: conduction	calculation of the sublimation rate under various process conditions
		mannitol		
(47)	Dyn	skim milk	vial bottom: gas conduction and radiation	calculation of the dynamic behavior of primary and secondary drying (*T*_P_, *w*_H2O_, *t*_Drying_) in trays
			product surface: gas conduction and convection	
(35)	Dyn	skim milk	application of specific heat flows	calculation of the dynamic behavior of primary and secondary drying (*T*_P_, *w*_H2O_, *t*_Drying_)
		bovine somatotropin		
(41)	Dyn	skim milk	vial bottom:gas conduction	calculation of the dynamic behavior of primary and secondary drying (*T*_P_, *w*_H2O_, *t*_Drying_)
			product surface: radiation	
(48)	Dyn	water	vial bottom: gas conduction	calculation of the influence of vial properties, position, filling level, and chamber pressure on heat transfer
			edge vials: additional radiation	
(42)	Dyn	water	vial bottom: gas conduction	calculation of the dynamic behavior of primary and secondary drying (*T*_P_, *w*_H2O_, *t*_Drying_)
			radiation on vial wall	
(33)	Dyn	5 w/v % sucrose	vial bottom: contact, gas conduction, and radiation	calculation of the dynamic behavior of primary drying (*T*_P_, *w*_H2O_, *t*_Drying_)
			product surface: radiation (stopper: insulation)	
(43)	Dyn	bovine serum albumin	vial bottom: contact and gas conduction	calculation of the dynamic behavior of primary and secondary drying (*T*_P_, *w*_H2O_, *t*_Drying_)
(49)	Dyn	5% sucrose,	vial bottom: contact, gas conduction, and radiation	process optimization
		10 mM Tris-HCl	product surface: radiation	
		4% PVP, 1% sucrose, 10 mM Tris-HCl		
(32)	Dyn	5% BSA, 0.1 M Tris-HCl	vial bottom: contact, gas conduction, and radiation	calculation of the dynamic behavior of primary drying (*T*_P_, *w*_H2O_, *t*_Drying_)
			product surface: radiation (stopper: insulation)	
			conduction through vial walls considered	
	Pse 1		vial bottom: convection and radiation	development of model-based optimization algorithms
	Pse 2		vial bottom: contact, gas conduction, and radiation	
			conduction through vial walls considered	
(34)	Dyn	skim milk	vial bottom: convection	calculation of the dynamic behavior of primary drying (*T*_P_, *w*_H2O_, *t*_Drying_)
			product surface: radiation	
(50)	Pse	5 w/w % sucrose	vial bottom: contact, gas conduction, and radiation	primary drying process development and optimization
				consideration of microcollapse
(51)	Pse	model formulation	vial bottom: contact, gas conduction, and radiation	process optimization including uncertainty analysis
(52)	Pse	5 w/v % mannitol	vial bottom: contact, gas conduction, and radiation	simultaneous K_v_ and R_p_ determination, process development, and scale-up
(5)	Dyn	25 g/L sucrose	vial bottom: contact, gas conduction, and radiation	calculation of the dynamic behavior of primary drying (*T*_P_, *w*_H2O_, *t*_Drying_)
(53)	Pse (batch)	3% sucrose	vial bottom: contact, gas conduction, and radiation	primary drying process development and optimization
	Pse (continuous)	3% lactose	radiation from IR emitters and surroundings	comparison between batch and continuous process
		3% mannitol		
(38)	Dyn	lactic acid bacteria, 200 g/L sucrose, 0.15 M NaCl	vial bottom: contact, gas conduction, and radiation	real-time process optimization
			product surface: radiation	
(24)	Pse	25 g/L sucrose	vial bottom: contact, gas conduction, and radiation	process optimization including uncertainty analysis
(54)	Pse	2 mM histidine, 10 g/L lyosozyme,	vial bottom: contact, gas conduction, and radiation	process optimization including uncertainty analysis
		5% 2-hydroxypropyl-ß-cyclodextrine,		
		0.03% polysorbate 80		
		2 mM histidine,		
		1 g/L lysozyme,		
		10% sucrose,		
		0.03% polysorbate 80		
(55)	Pse	water	vial bottom: contact, gas conduction, and radiation	*K*_v_ determination

a (Dyn: sorption-sublimation, Pse: pseudostationary, *T*_P_: product temperature, *w*_H2O_: residual moisture, and *t*_Drying_: drying times).

In this study, an instationary sorption-sublimation model is validated against experimental data. The moving boundary problem is addressed by mapping the sublimation front to the boundary of a fixed domain during the primary drying phase. The developed model, building upon the authors’ previous work, demonstrates novelty through its superior predictive fit, accuracy, and precision while also enabling efficient model parameter determination. A validation workflow of Sixt et al. has been used. Simulation results exhibit similar uncertainty compared to experimental data, with the deviation between them reduced to 3%. This constitutes a marked improvement over the 31.8% deviation observed with the pseudostationary model. The model is distinctly quantitatively validated and should now be used for the accelerated process design and advanced process control.

## 2 Materials and Methods

### 2.1 Product Mixture and Instruments

To prepare the sucrose solution, 25 g/L of d(+)-sucrose (>99.5%, p.a., Carl Roth GmbH + Co. KG, Karlsruhe, Germany) was dissolved in purified water (Arium Pro, Sartorius AG, Göttingen, Germany). The masses were measured using a laboratory scale LC 1200 S (Sartorius AG, Göttingen, Germany).

### 2.2 Freeze-Drying Equipment

The freeze-drying experiments were conducted using an Epsilon 2-6D LSCplus pilot freeze-dryer (Martin Christ Gefriertrocknungsanlagen GmbH, Osterode am Harz, Germany). A total of 2 mL of the product solution was filled into 6R injection vials (Martin Christ Gefriertrocknungsanlagen GmbH, Osterode am Harz, Germany) using an Eppendorf Research plus 0.5–5 mL pipet (Eppendorf AG, Hamburg, Germany). During the experiments, one of the three available shelves was fully loaded with 135 vials. Throughout the freeze-drying process, the product temperature was measured with “Wireless Temperature Measurement plus” (WTMplus) sensors (Martin Christ Gefriertrocknungsanlagen GmbH, Osterode am Harz, Germany) placed centrally on the vial bottom.

### 2.3 Model Validation

A model validation workflow from Sixt et al. is used to develop a model with sufficient accuracy and precision.^9^ First, the modeling tasks need to be defined to derive suitable model equations. Different decision criteria need to be met for successful model validation:

1. Verification:

- Detect gross errors through simulated one-parameter-at-a-time studies and design of experiments (DoEs).
- Use statistical evaluations to identify significant process parameters.

1. Accuracy and Precision:

- Experimental model parameter determination.
- Assess impact of parameter errors on the model through error propagation.
- Comparison with experimental data.

1. Sensitivity analysis:

- Visualize and compare statistically significant process parameters.

After all criteria are met, the process model is verified and quantitatively validated.

### 2.4 Freeze-Drying Experiments

The freeze-drying cycle was adapted from the literature.^56^ Initially, the shelf temperature is lowered to −45 °C and held for 2 h. Then, it is raised to −20 °C and held for 1 h before returning to −45 °C for a final hold of 2 h. During the freezing and annealing phases, the shelf temperature changes at a rate of 1 °C per minute. The primary drying conditions vary based on a fractional factorial design of experiments (DoEs) outlined in Table 2. It is used to identify and compare significant process parameters for the outcome of the primary drying process (temperature and end point) between experiment and simulation. The center point (CP) is repeated three times.

**Table 2:** Experimental DoEs for the Primary Drying Phase^a^

	*T*_s_ (°C)	*p*_c_ (mbar)	fill volume (mL)	ramp (°C/min)
++++	0	0.15	2	1
+–+–	0	0.05	2	0.2
–+–+	–25	0.15	1	1
++––	0	0.15	1	0.2
––––	–25	0.05	1	0.2
+––+	0	0.05	1	1
––++	–25	0.05	2	1
–++–	–25	0.15	2	0.2
CP	–12.5	0.1	1.5	0.6

a Reprinted from ref (26) under the Creative Commons Attribution (CC BY) license. Copyright 2022 Juckers.

In the second DoE parameters from secondary drying phase (shelf temperature, chamber pressure, and duration) are also varied to identify the critical process parameters that govern the residual moisture of the products (Table 3). A fill volume of 2 mL was used, and the CP is repeated three times.

**Table 3:** Experimental DoEs for Both Drying Phases^5^ (PD: Primary Drying, SD: Secondary Drying)^a^

	*p*_c,PD_	*T*_S,PD_	*p*_c,SD_	*T*_S,SD_	*t*_SD_
–+––+	0.076	–25	0.01	–10	6
+++++	0.2	–25	0.05	10	6
+++––	0.2	–25	0.05	–10	2
–+++–	0.076	–25	0.05	10	2
+–––+	0.2	–35	0.01	–10	6
–––+–	0.076	–35	0.01	10	2
+––+–	0.2	–35	0.01	10	2
––+–+	0.076	–35	0.05	–10	6
++–––	0.2	–25	0.01	–10	2
+–+++	0.2	–35	0.05	10	6
–+–++	0.076	–25	0.01	10	6
––+––	0.076	–35	0.05	–10	2
CP	0.138	–30	0.03	0	4

a Reprinted from ref (5) under the Creative Commons Attribution (CC BY) license. Copyright 2020 Klepzig.

### 2.5 Model Parameter Determination

A sorption-sublimation model has been developed to calculate the primary and secondary drying behavior of vial freeze-drying. To calibrate the model to the underlying freeze-dryer and material system, different parameters need to be determined.

The vial heat transfer coefficient *K*_v_ is determined with ice sublimation tests. 6R vials (Martin Christ Gefriertrocknungsanlagen GmbH, Osterode am Harz, Germany) are filled with water, and one shelf is fully loaded. The primary drying conditions are set the same as in the DoEs, and it is aborted after around 4 h. Selected vials are weighed before and after the drying phase, and the product temperature is measured with WTMplus (Martin Christ Gefriertrocknungsanlagen GmbH, Osterode am Harz, Germany). *K*_v_ is then calculated by
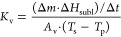
15with Δ*m* as the measured mass difference and Δ*t* as the primary drying duration.

The dry layer resistance *R*_p_ is determined by MTMplus (Martin Christ Gefriertrocknungsanlagen GmbH, Osterode am Harz, Germany). Every 10 min, the valve between the drying and ice condenser chambers is closed and the pressure increase is measured for a maximum of 30 s. The pressure rise data are analyzed with MTMplus Analysis software (Martin Christ Gefriertrocknungsanlagen GmbH, Osterode am Harz, Germany) to calculate *R*_p_. The relationship between dry layer resistance and dry layer height is described by
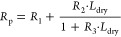
16with *R*_1_, *R*_2_, and *R*_3_ as constants to fit the equation to the experimental data and *L*_dry_ as dry layer height.

Modeling parameters are summarized in Table 4. ρ_I_ is calculated as the effective density together with the porosity.

**Table 4:** Model Parameters

parameter	description	value	unit
*A*_P_	inner cross-sectional area of the vial	3.14	cm^2^
*A*_v_	outer cross-sectional area of the vial	3.8	cm^2^
ρ_I_	dried layer density	30	kg/m^3^
ρ_II_^33^	frozen layer density	927	kg/m^3^
*c*_p,I_(33)	dried layer heat capacity	259.5	J/kg/K
*c*_p,II_(33)	frozen layer heat capacity	1940	J/kg/K
*c*_p,g_(33)	heat capacity gas in dry layer	2010	J/kg/K
*c*_w,s,eq_(33)	initial mass share of sorbed water	0.206	kg_water_/kg_solid_
λ_I_	dried layer heat conductivity	2.5·10^–4^	W/m/K
λ_II_^38^	frozen layer heat conductivity	2.4	W/m/K
σ	Stefan–Boltzmann constant	5.67·10^–8^	W/m^2^/K^4^
Δ*H*_subl_^33^	heat of sublimation of ice	2834.6	kJ/kg
Δ*H*_v_^33^	heat of vaporization of bound water	2499.6	kJ/kg
*R*	gas law constant	8314	J/mol/K
*M*_w_	molecular weight of water	18	kg/kmol
*E*	porosity	0.97	-
τ_ref_^38^	reference time constant	2.7·10^4^	s
*T*_ref_(38)	reference temperature in desorption	273.15	K
*E*_a_(38)	activation energy for desorption	4.27·10^4^	kJ/kg
*M*_g_(38)	constant of the GAB equation	0.0434	kg_water_/kg_solid_
*C*_g_(38)	constant of the GAB equation	7.4789	-
*K*_g_(38)	constant of the GAB equation	0.9827	-
*T*_S,PD_	primary drying shelf temperature	varied by DoE	K
*t*_PD_	primary drying duration	varied by DoE	h
*T*_S,SD_	secondary drying shelf temperature	varied by DoE	K
*t*_SD_	secondary drying duration	varied by DoE	h
*p*_c,PD_	chamber pressure primary drying	varied by DoE	Pa
*p*_c,SD_	chamber pressure secondary drying	varied by DoE	Pa

The residual moisture of the vials is determined with the gravimetric method. Emtpy, filled, and later dried vials are weighed with an analytical balance ABP 200-5DM (KERN & Sohn GmbH, Balingen, Germany). The mass of bound water was determined based on the known theoretical mass of the solid in the vial.

### 2.6 Software

Data from the freeze-drying cycles were collected by using LPCplus (Martin Christ Gefriertrocknungsanlagen GmbH, Osterode am Harz, Germany). Statistical analysis utilized JMP (JMP Inc., SAS Institute, Cary, NC), and Aspen Custom Modeler (Aspen Technology Inc., Bedford, MA) was used for simulations.

## 3 Results

### 3.1 Model Reformulation

**Figure 1:**
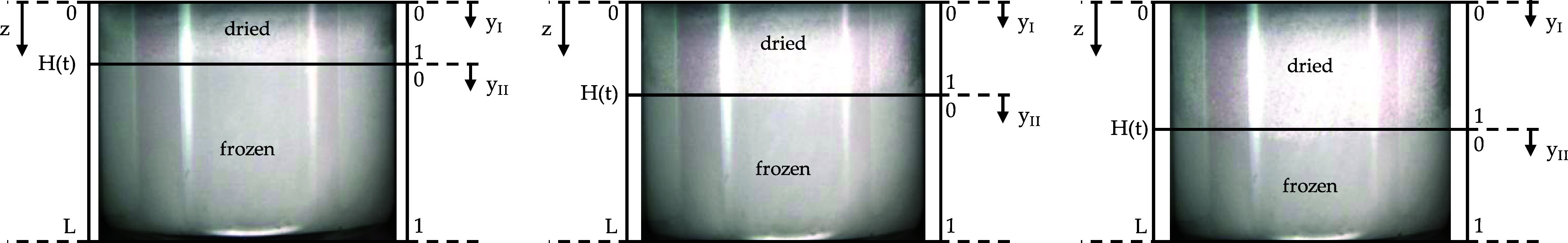
Display of the moving boundary during primary drying in dimensional and dimensionless coordinate system (*z* vs *y*).

The model equations describe a moving boundary problem, commonly referred to as the Stefan problem. Solving this equation system requires a spatial grid that evolves over time as the position of the sublimation front changes. To address this, a mathematical technique is applied to map the moving interface to a boundary of a fixed domain (Figure 1).

Sheehan and Liapis suggested a procedure, introducing two nondimensional variables.^41^ Each function and the derivatives are transformed according to the new coordinates. The dried region (layer I, 0 < *z* < *H*(*t*)) is now described by
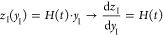
17and the frozen region (layer II, *H*(*t*) < *z* < *L*) by
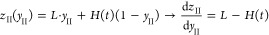
18where *y*_*I*_,*y*_II_∈[0, 1] are the new coordinates on the fixed domains, *L* is the fill height, and *H*(*t*) is the axial position of the sublimation interface.

Reformulating the model equations to the new fixed domain resolves the issue of the moving boundary; however, it introduces singularities. This is exemplified with the heat transfer equations for the dried
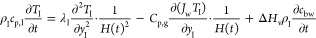
19and the frozen layers
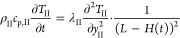
20

To prevent the singularity, a small dried layer needs to be present in the beginning and a small frozen layer in the end. The moving boundary is limited to the primary drying, as the secondary drying phase involves only the dried layer with all ice removed, allowing utilization of the dimensional model equations.

### 3.2 Accuracy and Precision

To validate the model, experimental data need to be compared to the simulation.^9^ The sorption-sublimation model can predict the product temperature of the vial. To achieve a validated model, the modeled results should have a deviation similar to that of the experiments. The model error is estimated through Monte Carlo simulations. Varied parameters and their range are summarized in Table 5. The model parameters are varied within their experimentally determined reproducibility. Edge and center vials are differentiated by the *K*_v_ value.

**Table 5:** Varied Parameter in Monte Carlo Simulations

parameter	values	unit
*V*_fill_	1.5 ± 0.045	mL
*p*_c_	0.1 ± 0.006	mbar
*T*_shelf_	–12.5 ± 1	°C
*K*_v_(26)	18.01 ± 3.8 (edge vial)	W/m^2^/K
	11.04 ± 1.2 (center vial)	W/m^2^/K
*R*_1_(26)	26,834 ± 7404	m/s
*R*_2_(26)	1.62 · 10^7^ ± 1.9 · 10^7^	1/s
*R*_3_(26)	42.76 ± 181.47	1/m

**Figure 2:**
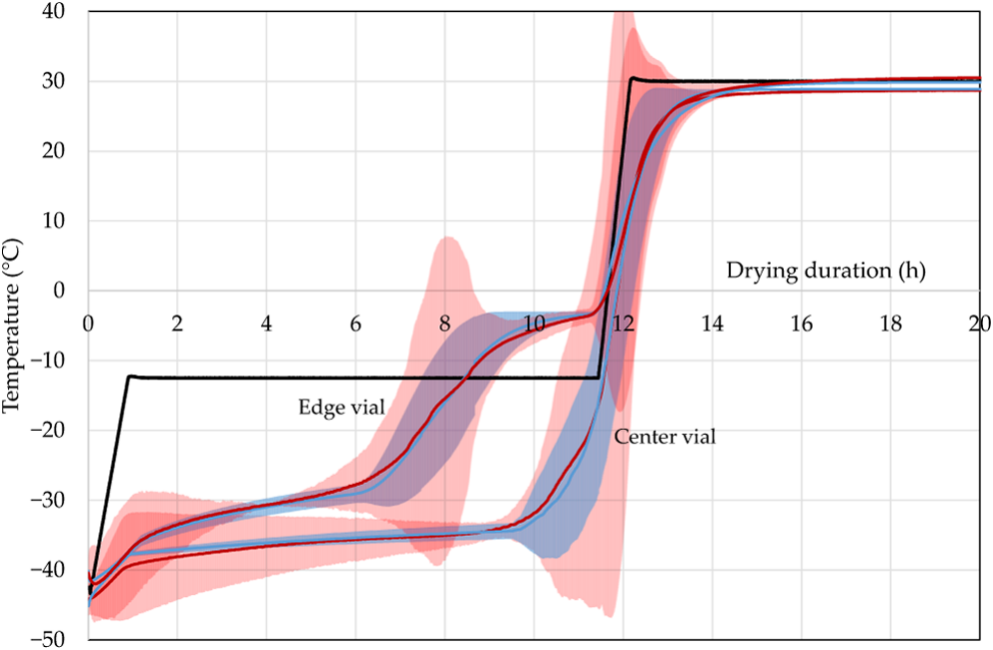
Experimental^26^ (red) vs simulated (blue) product temperature for an edge and center vial with shelf temperature (black) (*p*_c_ = 0.1 mbar, fill volume 1.5 mL). Reprinted in part from ref (26) under the Creative Commons Attribution (CC BY) license. Copyright 2022 Juckers.

In Figure 2, simulated results are plotted against experimental findings. The experiment was repeated three times for error calculation.

First, the results for the edge vial are compared. The initial temperature is −40.4 ± 2.2 °C for the experiments and −40.2 ± 0.1 °C for the simulation. After 2 h, it increases to −33.5 ± 4.4 °C (simulation: −33.7 ± 0.9 °C). Primary drying is finished after 7.1 ± 0.6 h with −23.1 ± 11.5 °C, and product temperature equals shelf temperature after 8.5 h for the experiments. The high deviation of the product temperature is caused by the difference in primary drying end points that create high temperature differences. In the simulation, all ice is removed after 7 ± 0.5 h at −24 ± 4.2 °C, and product temperature equals shelf temperature after 8.3 h.

At low shelf temperatures, radiation is a significant contribution to the heat transfer. The product temperature of the edge vial increases to −3 ± 0.5 °C (simulation: −3.1 ± 0.7 °C) after primary drying is finished. Average experimental product temperature during primary drying is −31.8 ± 4.3 °C (simulation: −32.1 ± 3.9 °C) and the relative error lies between 0.04 and 50.1% (simulation: 0.001 and 25%).

Secondary drying started around 11.4 h. The shelf temperature is further increased to desorb bound water from the formulation. The product temperature quickly rose to 25.2 ± 6.2 °C (simulation: 24.1 ± 2.6 °C) after 13 h until reaching the end temperature of 30.4 ± 0.1 °C (simulation: 29.8 ± 0.1 °C) at 20 h.

Next, the results for the center vial are presented. After 2 h, the experiments showed a product temperature of −38.3 ± 6.4 °C, while the simulation indicated −37 ± 0.5 °C. The temperature increases to −35.4 ± 2.5 °C (simulation: −35.1 ± 1 °C) after 7 h. Primary drying is finished after 10.3 ± 2.3 h (simulation: 10.7 ± 0.5 h) with a product temperature of −31.4 ± 6.6 °C in the experiments and −31.2 ± 6 °C in the simulation. The average primary drying product temperature in the experiment was found to be −36.7 ± 2.5 °C, compared to −36.1 ± 2 °C in the simulation. Regarding relative errors, the experiment showed a range from 1.6 to 21.1%, whereas the simulation exhibited a lower range of 0.1 to 13.5%. Due to the additional heat input through radiation, edge vials dry at higher temperatures than center vials.

During secondary drying, the temperature in the experiment rose to 23.8 ± 6 °C, while the simulation predicted a temperature of 25.4 ± 3.6 °C, after 13 h. Finally, after 20 h, the experimental measurement was 28.7 ± 0.2 °C, with the simulation closely matching at 28.8 ± 0.1 °C. The data reveal a good agreement between the mean of the experimental and simulated temperature profiles.

**Figure 3:**
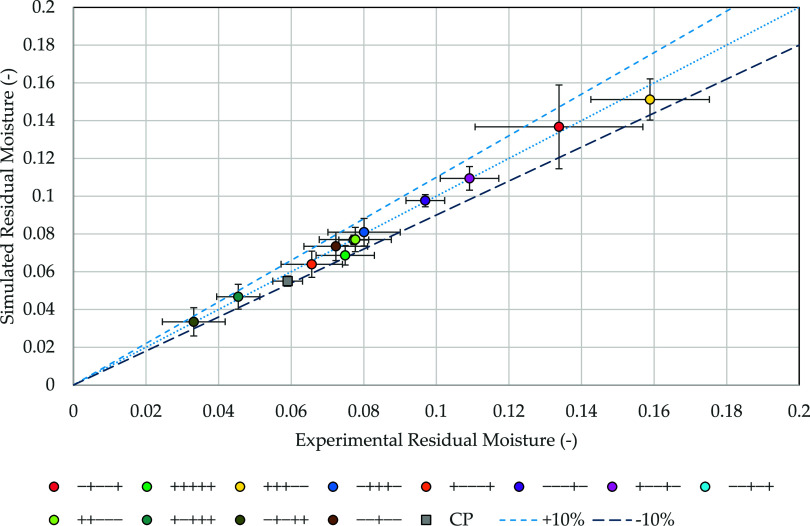
Parity plot of simulated and experimental residual moisture.

Next, the results of the sorption-sublimation model regarding the residual moisture during secondary drying are analyzed and compared (Figure 3).

The highest residual moisture was found at the experimental points + ++-- and -+--+ with 15.9 ± 0.02 and 13.4 ± 0.02%. The predicted values deviate 4.8 and 2.1% from the experimental findings with a comparable uncertainty (+++--: 10.3% (experiment), 7.2% (simulation); -+--+: 17.3% (experiment), 16.2% (simulation)). The lowest values were determined at -+-++ and + -+++. Here, the shelf temperature and secondary drying duration are increased, facilitating the desorption process. Experimental values of 3.3 ± 0.8 and 4.5 ± 0.6% are determined, while the simulation predicts 3.3 ± 0.7 and 4.6 ± 0.07%. The residual moisture at the center point of the DoEs (Table 3) was experimentally determined to 5.59 ± 0.41%. The simulation results deviated by 8% from this value, with both the experimental and the simulated outcomes showing a relative error of 6.9%. Again, good agreement was shown between simulation and experiment.

### 3.3 Sensitivity Analysis

Next, the results of the DoEs are fed into a statistical program and analyzed for statistically significant process parameters. At first the primary drying DoE is analyzed and compared with the results from experiments and simulation with a pseudostationary model from another study.^26^

**Figure 4:**
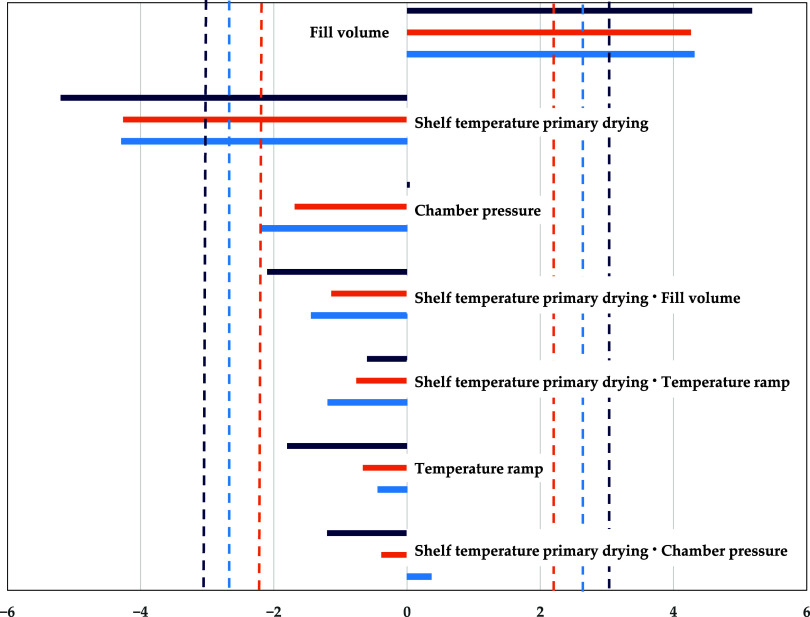
Pareto diagram of standardized effects for primary drying end point (light blue: experiment,^26^ orange: PSE,^26^ dark blue: sorption-sublimation model, dashed line: significance threshold). Reprinted in part from ref (26) under the Creative Commons Attribution (CC BY) license. Copyright 2022 Juckers.

To evaluate the interaction and strength of various process parameters in simulations and experiments, Pareto plots are employed. They identify statistically significant process parameters and their impacts on process performance. Both the experimental data and simulations indicate that fill volume and shelf temperature are the most influential parameters for the primary drying duration (Figure 4). Fill volume correlates positively and shelf temperature negatively. An increased fill volume leads to a higher amount of water that needs to be removed during the primary drying step, prolonging the process, while an increased shelf temperature accelerates the process by supplying more energy to the endothermic sublimation.

**Figure 5:**
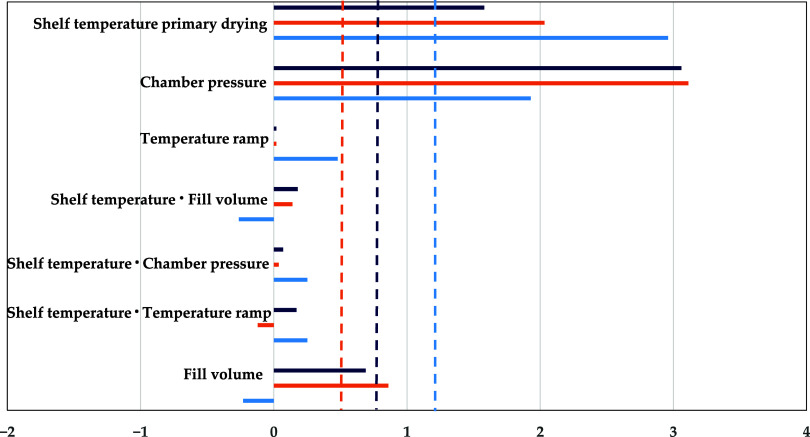
Pareto diagram of standardized effects for primary drying product temperature (light blue: experiment,^26^ orange: PSE,^26^ dark blue: sorption-sublimation model, dashed line: significance threshold). Reprinted in part from ref (26) under the Creative Commons Attribution (CC BY) license. Copyright 2022 Juckers.

For the primary drying product temperature, the results are shown in Figure 5. Shelf temperature and chamber pressure are significant parameters in both experiments and simulations, as an increase in either raises the heat supplied to the vial that consequently raises the product temperature. In the pseudostationary simulations, fill volume also emerges as a significant parameter. However, this could not be evidenced with the experiments. The sensitivity of the instationary sorption-sublimation model can accurately reflect experimental results.

**Figure 6:**
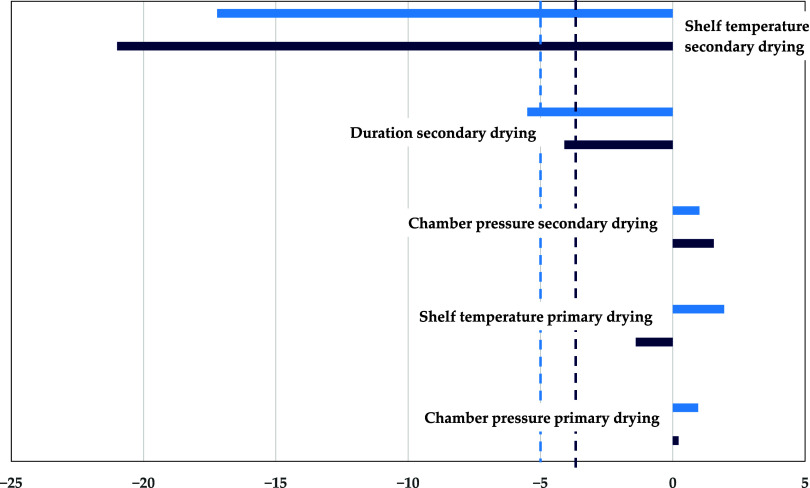
Pareto diagram of standardized effects for residual moisture content (light blue: experiment,^5^ dark blue: sorption-sublimation model, dashed line: significance threshold). Reprinted in part from ref (5) under the Creative Commons Attribution (CC BY) license. Copyright 2020 Klepzig.

In Figure 6, the significant parameters for residual moisture are identified. Shelf temperature and duration of secondary drying are significant. Desorption is an equilibrium process that requires a sufficient duration to achieve low residual moisture. Increasing the shelf temperature supplies more energy for desorption and increases the product temperature to allow accelerated desorption for low residual moisture levels.

The instationary simulations demonstrate higher precision and accuracy, effectively describing parameter interactions and strengths similar to those of the experiments. Consequently, this modeling approach can replace experiments for developing and optimizing freeze-drying process conditions.

### 3.4 Validation with New Freeze-Drying Process

**Figure 7:**
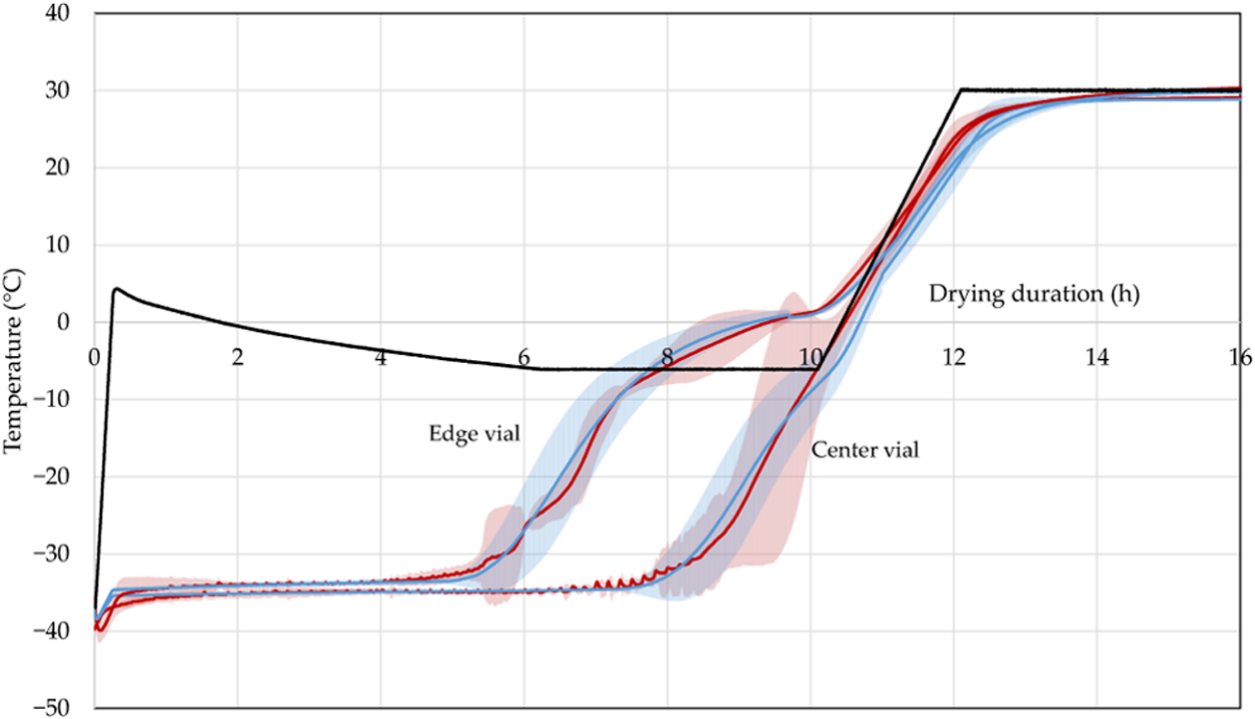
Experimental^24^ (red) vs simulated (blue) product temperature for an edge and center vial with optimized process conditions (controlled nucleation at −8 °C, *p*_c_ = 0.15 mbar, and fill volume 2 mL). Reprinted in part from ref (24) under the Creative Commons Attribution (CC BY) license. Copyright 2024 Juckers.

Next, the product temperature profile of an optimized recipe is shown in Figure 7. The primary drying conditions have been optimized by the pseudostationary process model.^24^ Here, the shelf temperature is dynamically changed throughout the drying phase. The simulation error is estimated with Monte Carlo simulations. The parameter range is shown in Table 6. The model parameters are varied within their experimentally determined reproducibility. Edge and center vials are differentiated with the *K*_v_ value.

**Table 6:** Varied Parameters for Monte Carlo Simulations

parameter	values	unit
*V*_fill_	2 ± 0.06	mL
*p*_c_	0.15 ± 0.009	mbar
*T*_shelf_	±1	°C
*K*_v_(24)	17.02 ± 1.3 (edge vial)	W/m^2^/K
	13.87 ± 1 (center vial)	W/m^2^/K
*R*_1_(24)	34,037 ± 2632	m/s
*R*_2_(24)	7.41 · 10^6^ ± 8.9 · 10^6^	1/s
*R*_3_(24)	0	1/m

The initial temperature of an edge vial is −35.7 ± 0.8 and −39.7 ± 0.4 °C for a center vial. The initial temperature of the simulation deviates 3.8% for an edge and 6.7% for a center vial. The values are −37.1 ± 0.1 and −37.2 ± 0.1 °C, respectively. After 2 h, the edge vials have a temperature of −34 ± 0.4 °C (simulation: −34.1 ± 0.4 °C). This is 3.5% higher than the center vials with −35.2 ± 0.6 °C (simulation: −35.1 ± 0.1 °C). Due to the higher product temperature, the sublimation of edge vials is finished 30% earlier than center vials after 5.6 ± 0.9 h (simulation: 5.8 ± 0.4 h). The final product temperature of the edge vial is −30.4 ± 6.3 °C. The simulation deviates 2.25% from this value with −31.1 ± 3.5 °C. Center vials have a temperature of −34.8 ± 0.4 °C (simulation: −34.8 ± 0.2 °C). After 8.3 ± 0.9 h (simulation: 8.1 ± 0.7 h), the center vials are finished with sublimation. The calculated product temperature deviates 2% from the experimental (experiment: −31.2 ± 4.2 °C; simulation: −30.6 ± 5.1 °C).

After 10 h, the primary drying of all vials is finished, and the shelf temperature is further increased to facilitate desorption. For the edge vial, an experimental product temperature of 1.3 ± 0.2 °C could be measured, and a temperature of 1 ± 0.1 °C was calculated. Experimental and simulated product temperature of the center vial show a deviation of 5% (experiment: −7.5 ± 8.9 °C, simulation: −34.8 ± 0.8 °C). Here, again the deviation in primary drying end points leads to high experimental uncertainties. For both vial types, the product temperature increases quickly. For edge vials to 22.8 ± 1.5 °C after 12 h and 30.3 ± 0.4 °C after 16 h and for center vials from 23.8 ± 2.1 to 29.1 ± 0.3 °C. The simulation deviates 10% after 12 h (20.7 ± 1.5 °C) and 2% after 16 h (29.8 ± 0.1 °C). The deviation for the center vial is of the same magnitude.

**Figure 8:**
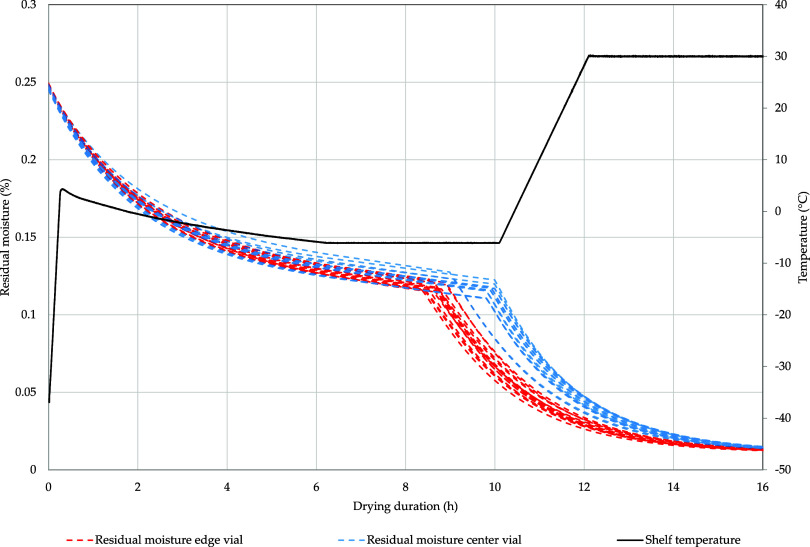
Simulated residual moisture and product temperature over drying time for the edge and center vials.

Together with the product temperature, the residual moisture of the dried layer can be calculated throughout both drying phases (Figure 8). Due to the low initial product temperature, all vials start with a residual moisture of 24.7 ± 0.1% that gradually decreases during the drying phases. Initially, the moisture loss is similar but as soon as primary drying is finished for edge vials and the product temperature increases the desorption process is accelerated. As the shelf temperature increases, the desorption process is accelerated for all vials. As they reach similar product temperatures during secondary drying, differences in residual moisture are reduced.

**Figure 9:**
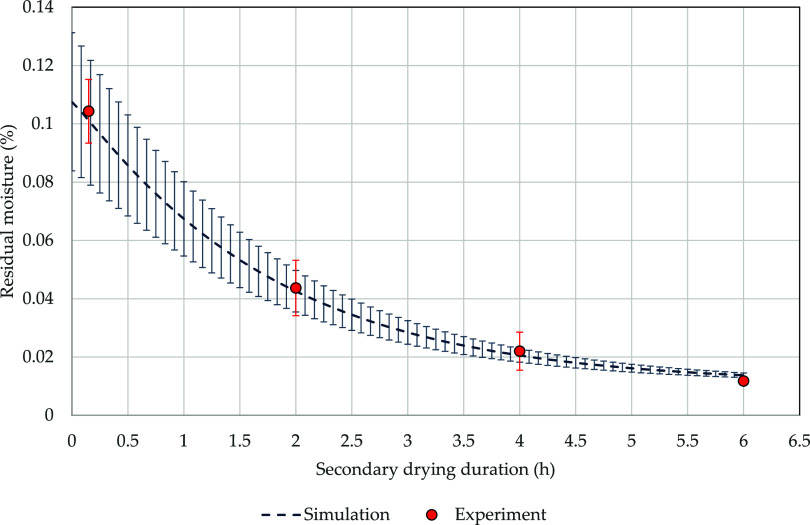
Experimental (red) vs simulated (blue) residual moisture of the product during the secondary drying stage over time.

For easier comparison of the residual moisture, the experiments are compared to the simulated residual moisture over the secondary drying duration (Figure 9). At the beginning, after 0.15 h, the experimentally measured residual moisture is 10.4 ± 1.1%, while the simulation results in 10 ± 2.1%. The relative error between the two values is 21.4% which is higher than the experimental uncertainty of 10.4%. Here, the difference in residual moisture between edge and center vials is high.

After 2 h, the final shelf temperature of 30 °C is reached. Here, the experimental residual moisture decreases to 4.4 ± 0.9%, while the simulation yields 4.3 ± 0.7%. Holding the final shelf temperature for 2 h (4 h total secondary drying time) decreases the experimental residual moisture to 2.2 ± 0.7%, while the simulation deviates 6.8% from this value. After 6 h, the secondary drying phase is finished. The experimental residual moisture reaches 1.2 ± 0.1%, while the simulation predicts a value of 1.3 ± 0.1%. The relative error of 12.1% is similar to the experimental uncertainty of 11.9%, evidencing precision and accuracy of the model predictions.

## 4 Discussion

In this work, a monodimensional sorption-sublimation model was validated to predict the effect of process parameters on the drying outcome. The moving boundary problem was solved by mapping it to a new dimensionless, fixed coordinate system. Singularities caused by the reformulation of the model equations are mitigated by using a small dried layer in the beginning and a small frozen layer at the end of primary drying. The precision and accuracy of the model were evidenced by comparing experimental and simulated results.

During the first DoE, only the conditions of the primary drying are changed to assess the impact of these parameters on the simulation results. The center point (*T*_shelf_: – 12.5 °C, *p*_c_: 0.1 mbar, fill volume: 1.5 mL) has been repeated three times to assess the experimental error. During primary drying, the average product temperature of an edge vial is −31.8 ± 4.3 °C which deviates 1% from the calculated value of −32.1 ± 3.9 °C. This deviation is smaller than the experimental error of up to 50%. The simulation is more precise with a maximum deviation of 25%. Similar results can be found with colder center vials. An average experimental product temperature of −36.7 ± 2.5 °C deviating 1.7% from the predicted value of −36.1 ± 2 °C. This deviation is smaller than the experimental uncertainty of 1.6 to 21.1%, evidencing the accuracy and precision of the simulation.

Comparing the results of this simulation with the results from a pseudostationary model from another study of the authors^26^ shows the advantage of this model. For an edge vial, an average product temperature of −34.9 ± 1.9 °C could be calculated, deviating 9.4% from the experimental value. Especially, the product temperatures at the end of primary drying show a high difference (experiment: −23.1 ± 11.5 °C, sorption-sublimation: −24 ± 4.2 °C, pseudostationary: −33.8 ± 4.3 °C). The sorption-sublimation model is superior in calculating drying conditions with high amounts of radiational heat, where heat accumulation in the frozen phase should not be neglected. This is made evident by comparing the results of the center vial. Here, an average product temperature of −36 ± 1.5 °C could be calculated, which deviates by 1.9% from the experiments. The difference between product temperatures at the primary drying end point could also be reduced (experiment: −31.4 ± 6.6 °C, sorption-sublimation: −31.2 ± 6 °C, pseudostationary: −33.4 ± 2.4 °C). It is also sensitive to significant process parameters and can reflect the experimental findings more effectively, describing parameter interactions and strengths accordingly.

The following secondary drying can be described only by the sorption-sublimation model. During secondary drying, the shelf temperature is further increased to facilitate desorption of bound water to achieve low residual moistures. The predicted product temperature differs from the experimental value by 2–10%. Uncertainties are comparable, 0.35–7.3% versus 1–8.9%. The exact determination of the product temperature allows for accurate prediction of the residual moisture. Experiment and simulation show similar error margins of 3.3 to 26.1%, and the simulation only deviates 0.2–8.3% from the experimental value.

The sorption-sublimation model exhibits the same sensitivities as those in the experiments. The primary drying product temperature is statistically dependent on shelf temperature and chamber pressure, while the primary drying end point is additionally influenced by the fill volume. Shelf temperature and drying duration during secondary drying are critical for the final residual moisture content. The simulations demonstrate high precision and accuracy, effectively describing the parameter interactions and their magnitudes. Consequently, this modeling approach can serve as a reliable alternative to experiments for developing and optimizing freeze-drying process conditions.

To further validate the transient process model, drying conditions beyond the previously tested experimental values are applied. The optimized primary drying conditions are determined using the pseudostationary model. Here, the shelf temperature changes dynamically over time. The experiment showed an average product temperature of −33.9 ± 1.4 °C for an edge vial and −34.9 ± 1 °C for a center vial. The sorption-sublimation model predicts values of −34 ± 0.9 or −34.8 ± 0.8 °C. The results deviate 0.3% for both vials. The experimental uncertainty lies between 2.0 and 20.7% for an edge vial and 0.03 and 13.6% for a center vial. The simulation shows smaller uncertainty than the experiments with 0.01–11.3% for an edge vial and 0.01–9.9% for a center vial. Again, this evidenced the precision and accuracy of the developed sorption-sublimation model. The pseudostationary model calculates the average product temperature to −32.6 ± 2.1 °C for an edge vial and −33.9 ± 2.4 °C for a center vial, deviating 3.8% from the edge vial and 2.9% from the center vial. With optimized process conditions, the accuracy of the pseudostationary model can be improved, especially for the edge vial. However, the sorption-sublimation model is still more precise.

During secondary drying, the shelf temperature is increased to 30 °C over 2 h with a holding time of 4 h. The residual moisture decreases over time, starting at 10.4 ± 1.1% experimentally and 10 ± 2.1% in the simulation after 0.15 h, with a relative error of 4.1%. After 2 h, the experimental moisture drops to 4.4 ± 0.9%, while the simulation predicts 4.3 ± 0.7%. At the end of secondary drying, the experimental moisture reaches 1.2 ± 0.1%, and the simulation predicts 1.3 ± 0.1%, with a relative error of 12.2% similar to the experimental uncertainty of 11.9%, confirming the accuracy and precision of the sorption-sublimation model.

The big advantage of the pseudostationary model against the sorption-sublimation model is its simplicity. The model does not require complex solution algorithms or specialized software making the implementation in Excel possible.^57^ This simplicity comes with the drawback of losing precision and accuracy especially at process parameters where radiation cannot be neglected. Here, the sorption-sublimation model is far superior by allowing precise calculations of the process. Since both models utilize the same model parameters *K*_v_ and *R*_P_, they should not be seen as competitors. Instead, they should be considered complementary and used in combination to fully leverage their respective strengths. Pseudostationary model to initiate process development and then use the sorption-sublimation model to further tune the process parameters for an optimized recipe.

The selection of PAT tools for the secondary drying phase is limited, making it challenging to compare the real-time state with in silico calculations in a digital twin. Additionally, the model parameter determination for the secondary drying is time-consuming, requiring the determination of the water isotherms and drying kinetics with specialized equipment.^33,36,58,59^Product temperature can be determined with temperature sensors. However, residual moisture determination is more sophisticated. Pressure rise test,^60,61^ tunable diode laser absorption spectroscopy (TDLAS),^61,62^ near-infrared spectroscopy (NIR),^63−65^ and mass spectrometry (MS)^66^ are available. Residual moisture is a critical product quality attribute and is important for regulatory approval and quality assurance. Adoption of NIR has been reported for spin freeze-drying.^67^ Implementation in batch freeze-drying production scale is hard due to the size of the sensors and the need for optical fibers, limiting the positional possibilities and compatibility with loading and unloading systems.^68,69^ To overcome these practical obstacles, the proposed model can be used as a soft sensor for operational parameter decisions within the design and control space defined a priori during QbD-based process development.

The sorption-sublimation model shows a very high accuracy and precision in predicting the process behavior of vial lyophilization. The deviation between experiment and simulation was reduced to 3.9% compared to 42.2% for the pseudostationary model at the end of primary drying for edge vials (CP DoE 1, experiment: −23.1 ± 11.5 °C, Dyn: −24 ± 4.2 °C, Pse: −32.9 ± 2.2 °C^26^). For autonomous operation using advanced process control methods, the proposed model needs to be overlaid with numerical optimization routines. However, the question of which method is the most efficient is the subject of research that is currently required and will be continued in the future.

## Abbreviations

QbD: quality by design
PAT: process analytical technology
WTMplus: wireless temperature measurement plus
TDLAS: tunable diode laser absorption spectroscopy
NIR: near-infrared spectroscopy
MS: mass spectrometry
